# Editorial: Surgical treatment of spinal infections: management of spondylodiscitis and implant-associated vertebral osteomyelitis

**DOI:** 10.3389/fsurg.2024.1455162

**Published:** 2024-07-23

**Authors:** Siegmund Lang, Jonathan Neuhoff, Christoph Hohenberger, Sonja Häckel

**Affiliations:** ^1^ Department of Traumasurgery, University Hospital Regensburg, Germany; ^2^ Center for Spinal Surgery and Neurosurgery, BG Unfallklinik, Frankfurt am Main, Germany; ^3^ Department of Neurosurgery, Regensburg University Hospital, Germany; ^4^ Department of Orthopaedic Surgery and Traumatology, Inselspital, Bern University Hospital, University of Bern, Bern, Switzerland

**Keywords:** spinal infection, spondylodiscitis, vertebral osteomyelitis, implant-associated infection, spine, surgery

**Editorial on the Research Topic** Surgical treatment of spinal infections: management of spondylodiscitis and implant-associated vertebral osteomyelitis

Spinal infections, particularly pyogenic spondylodiscitis, and implant-associated vertebral osteomyelitis, continue to pose significant clinical challenges despite advances in diagnostic and therapeutic strategies. The rising incidence of these infections, coupled with the aging population and increasing number of spinal procedures, necessitates a robust focus on prevention, accurate diagnosis, and effective management. This special issue of Frontiers in Surgery compiles cutting-edge research and clinical insights to optimize the management of these debilitating conditions. Accurate diagnostics are essential for early detection and appropriate intervention in spinal infections. However, the variability in sensitivity and specificity across diagnostic modalities remains a challenge. Identifying the causative pathogen is crucial for targeted antibiotic therapy, yet pathogen-negative cases complicate the diagnostic process. The role of advanced imaging techniques and the evaluation of novel biomarkers are critical areas of ongoing research. The decision-making process for surgical intervention in spinal infections requires careful consideration of patient-specific and pathogen-related factors. From minimally invasive procedures to extensive surgical stabilization, selecting the optimal surgical strategy is pivotal for improving patient outcomes.

We extend our sincere gratitude to the authors for their significant contributions to this special issue. Their dedication and expertise in advancing our understanding of spinal infections have been invaluable. We also thank the reviewers for their thorough evaluations and insightful feedback, which have ensured the high quality and scientific rigor of the manuscripts. Lastly, we appreciate the editorial team's unwavering support and guidance, which have been crucial to the success of this special issue, maintaining the highest standards of scholarly publication.

This special issue provides a platform for sharing novel insights into the prevention, diagnosis, decision-making, and treatment strategies for spinal infections. It emphasizes diagnostic methods, factors influencing clinical outcomes related to patients and pathogens, and both established and innovative surgical techniques. The following manuscripts contribute to this special issue:

1. Extensive spinal epidural abscess caused by *Staphylococcus epidermidis*: A case report and literature review

This case report and review by Yang-wei Pi et al. highlights the importance of early diagnosis and combined surgical and antibiotic treatment for managing extensive spinal epidural abscesses caused by multidrug-resistant *Staphylococcus epidermidis*.

2. The one-stop-shop approach: Navigating lumbar 360-degree instrumentation in a single position

This pilot series by Maximilian Schwendner et al. demonstrates that navigated lumbar dorsal and lateral instrumentation for pyogenic spondylodiscitis in a single surgery and positioning is feasible and safe, potentially reducing intraoperative radiation exposure and improving surgical outcomes.

3. Clinical phenotyping of spondylodiscitis and isolated spinal epidural empyema: a 20-year experience and cohort study

This retrospective cohort study by Mido Max Hijazi et al. reveals distinct clinical phenotypes and outcomes of spondylodiscitis and isolated spinal epidural empyema (ISEE), with the latter showing a more favorable disease course and lower complication rates.

4. Clinical and surgical outcome in patients with cervical spondylodiscitis: a single-center retrospective case series

The analysis of 24 patients with cervical spondylodiscitis by Stefan Motov et al. identifies that despite surgical and antibiotic treatment, the disease often results in permanent neurological damage or fatal outcomes, particularly in complicated cases.

5. Prevention of implant-associated spinal infections: the GAID-protocol

This study by Joanna Maria Przybyl et al. demonstrates the efficacy of the GAID-Protocol (Gloves, Antiseptics, Implants, and Drainage-use in large wounds) in reducing severe wound complications and implant-associated spinal infections, suggesting that adherence to this protocol can significantly improve patient outcomes.

6. The impact of concomitant infective endocarditis in patients with spondylodiscitis and isolated spinal epidural empyema

Mido Max Hijazi et al. characterize the clinical phenotypes of spinal infections with concomitant infective endocarditis, highlighting the high sensitivity of the modified Duke criteria for detecting endocarditis in these patients.

7. Midterm survival and risk factor analysis in patients with pyogenic vertebral osteomyelitis: a retrospective study

This retrospective analysis by Melanie Schindler et al. identifies significant risk factors for mortality in patients with pyogenic vertebral osteomyelitis, emphasizing the need for early identification and management of these risk factors to improve survival rates.

8. Selection of treatment strategies for lumbar *Brucella spondylitis*: a retrospective clinical study

This comparative analysis of treatment strategies for lumbar *Brucellar spondylitis* by Changhao Liu et al. demonstrates that surgical treatment achieves early recovery goals more effectively than pharmacological treatment, with individualized approaches being essential for optimal patient outcomes.

The special issue identified several critical risk factors associated with poor outcomes in spinal infections, providing essential insights for selecting the most appropriate treatments for individual patients. These findings may pave the way for integrating big data approaches to enhance patient-specific treatment strategies. The continuous advancements in surgical techniques, aimed at minimizing surgical stress and reducing morbidity in this vulnerable patient population, are highlighted. Collectively, these efforts signify substantial progress in managing and treating spinal infections, with a clear focus on reducing the overall burden of these conditions on patients’ lives. The collaborative efforts of the researchers and clinicians involved are evident in the innovative approaches and valuable insights shared, all aimed at improving patient outcomes and care. The presented research has deepened our understanding of spinal infections and has laid the groundwork for future collaborative advancements.

